# Exploring Health Care Professionals’ Perspectives on Education, Awareness, and Preferences for Digital Educational Resources to Support Transgender, Nonbinary, and Intersex Care: Interview Study

**DOI:** 10.2196/67993

**Published:** 2025-03-06

**Authors:** Sravya Katta, Nadia Davoody

**Affiliations:** 1 Health Informatics Centre Department of Learning, Informatics, Management, and Ethics Karolinska Institutet Stockholm Sweden

**Keywords:** health care professionals, transgender, nonbinary, and intersex, communication challenges, systematic barriers, information and communication technology

## Abstract

**Background:**

Health care professionals often face challenges in providing affirming and culturally competent care to transgender, nonbinary, and intersex (TNBI) patients due to a lack of understanding and training in TNBI health care. This gap highlights the opportunity for tailored educational resources to enhance health care professionals’ interactions with TNBI individuals.

**Objective:**

This study aimed to explore health care professionals’ perspectives on education and awareness of health issues related to TNBI individuals. Specifically, it aimed to identify their needs, challenges, and preferences in accessing and using digital educational resources to enhance their knowledge and competence in providing inclusive and effective care for this population.

**Methods:**

A qualitative research approach was used in this study. In total, 15 health care professionals were recruited via convenience sampling to participate in semistructured interviews. Thematic analysis was applied to identify recurring codes and themes.

**Results:**

The study identified several themes and subthemes related to gender diversity awareness, inclusive communication and understanding the needs of TNBI individuals, societal and structural challenges, regulatory gaps in training and support infrastructure, education and training needs for health care professionals on TNBI care, educational resources and training tools for TNBI care, challenges and design considerations for eHealth tools integrations, and evaluating eHealth impact. Participants identified communication barriers, the need for health care providers to use inclusive language, and gaps in both health care system infrastructure and specialized training for gender-affirming care. In addition, participants expressed a need for comprehensive education on transgender and nonbinary health issues, resources for mental health professionals, user-friendly design, and accessibility features in eHealth tools.

**Conclusions:**

The study revealed substantial deficiencies in health care professionals’ knowledge of gender diversity, cultural competency, and the importance of inclusive communication. Addressing the identified barriers and challenges through targeted interventions, such as providing training and support for health care professionals, investing in user-friendly design and data security, and promoting cultural competence in TNBI health care, is essential. Despite integration challenges, eHealth tools have the potential to improve patient–health care professional relationships and access to care.

## Introduction

### Background

The term transgender refers to individuals whose gender identity or expression differs from the sex they were assigned at birth [1]. The concept has expanded over time to include a wide range of gender identities, such as transmen, transwomen, nonbinary individuals, and those who are gender nonconforming [2,3]. Nonbinary individuals may not exclusively identify as male or female. Their gender identity can be fluid, agender, or fall outside the binary spectrum [1,4]. The transgender community is highly diverse, and the understanding of transgender identity varies across different cultures [2]. Intersex individuals are those whose physical sex characteristics do not conform to the traditional binary classification of bodies as strictly male or female [4]. Previous epidemiological and clinic-based investigations have suggested that approximately 0.1% to 2% of the population identifies as transgender or with other noncisgender identities [5-7].

The literature reveals that transgender, nonbinary, and intersex (TNBI) individuals experience disproportionate levels of human rights violations and adverse health outcomes, largely attributed to intersecting forms of social marginalization and legal exclusion. Particularly transgender individuals, especially those from minority ethnic groups, are disproportionately impacted by gender-based hate crimes [8]. In addition, TNBI individuals face multiple challenges in accessing adequate health care services [9-12]. The challenges faced by TNBI individuals in accessing health care services are often rooted in systemic barriers that perpetuate stigma, discrimination, and lack of understanding. These systemic barriers contribute to disparities in health care access, quality, and outcomes for these individuals [9-12]. These barriers may include policies that fail to recognize their gender identity and social factors such as discrimination and stigma from health care providers [9-13]. TNBI individuals often face a range of health disparities, largely driven by a lack of awareness regarding their unique health needs. These disparities manifest in higher rates of mental health issues, such as depression and anxiety, as well as increased risks of sexually transmitted infections, including HIV, and other diseases such as cancer, smoking-related conditions, and cardiovascular disease [14,15]. The elevated risk of acquiring sexually transmitted infections among TNBI individuals is influenced by factors such as limited access to comprehensive sexual health education, prevention measures, and adequate preventive care [16]. Furthermore, TNBI individuals often face significant barriers to accessing gender-affirming health care, which can lead to poorer overall health outcomes [13].

In addition to these health care challenges, TNBI individuals frequently experience widespread stigma, discrimination, and prejudice in various facets of life, including employment, education, housing, and health care. This systemic stigma surrounding gender diversity and nonconformity creates a hostile environment for TNBI individuals even within health care systems [6,17-20]. In a sexual health seminar held in Minnesota, a sample of 181 transgender participants revealed that 66% reported experiencing discrimination based on their gender identity or presentation [21]. Consequently, the denial of essential health care services can intensify feelings of dysphoria and distress among transgender individuals. While TNBI individuals face significant challenges in accessing care, health care professionals also encounter numerous obstacles that hinder their ability to provide effective support and services to these individuals.

### Challenges Faced by Health Care Professionals

Health care professionals play a crucial role in caring for patients, acting as key facilitators of essential health care services and resources. This highlights the responsibility of health care providers to promote the health and overall welfare of the populations including TNBI [22]. However, health care professionals face challenges in treating and communicating with TNBI individuals, as many of them receive minimal or no training during their medical education and professional development regarding hormone therapy, gender-affirming surgeries, and mental health support—key aspects that are aimed at aligning an individual’s physical appearance and gender identity with their affirmed gender during medical education and professional development [10,23].

In addition, health care professionals often encounter challenges when interacting with TNBI individuals, including issues related to cultural competency, communication barriers, and a lack of knowledge about appropriate care practices [24]. These challenges can result in disparities in health care access and quality for TNBI individuals, leading to negative health outcomes and experiences of discrimination [25]. Without adequate training, health care professionals may lack the necessary knowledge and skills to provide competent and affirming care to TNBI patients. This can result in misdiagnosis and inappropriate treatment for patients [26]. Moreover, health care professionals who are unfamiliar with the specific health needs of TNBI individuals may inadvertently overlook important aspects of care and may fail to provide appropriate interventions. Increasing awareness and understanding of TNBI health issues through education, professional development, and exposure to diverse patient populations can help health care professionals better meet the needs of their TNBI patients [27].

In this study, we focused on 4 countries—Sweden, India, the United Kingdom, and the United States—due to their diverse sociocultural context, legal frameworks, and health care systems. This selection provides valuable insights into the varying approaches to TNBI health care and medical education within each country, which allows for a broad examination of how medical education on TNBI individuals can be improved globally. Sweden’s progressive health care policies and a strong emphasis on human rights, including TNBI rights, make it an ideal setting to explore advanced practices and identify areas for improvement. India, with a complex sociocultural landscape and a vast and diverse population, presents unique challenges and opportunities in providing health care to TNBI individuals. Understanding health care professionals’ perspectives in India can reveal the specific needs and barriers faced by TNBI individuals in a resource-limited setting. The United Kingdom has recently experienced significant sociopolitical changes affecting health care policies for TNBI individuals. The United States’ diverse health care environment regarding TNBI care, with some states enacting progressive policies and others imposing restrictions on gender-affirming treatments, offers insights into the challenges and successes in providing care to TNBI individuals in such varied regulatory settings. By understanding these varied contexts, we aimed to identify gaps in education and awareness as well as potential best practices to address health care professionals’ needs, challenges, and preferences in accessing and using educational resources. This insight will help enhance their knowledge and competence in providing inclusive and effective care for TNBI individuals. The medical education systems in each of these countries present unique structures but share a common challenge: a lack of formal education on TNBI health care needs. In Sweden, despite its progressive stance on gender equality, medical curricula still rarely address the specific health care concerns of gender-diverse individuals. India’s medical education, influenced by traditional values and diverse cultural perspectives, similarly lacks comprehensive training on gender diversity, despite growing awareness of TNBI rights [28]. The United Kingdom and the United States have made strides in addressing health care inequalities, yet many health care professionals report limited exposure to TNBI health topics during their training [29,30].

### The Importance of Educational Tools and the Role of Information and Communication Technology

Lack of necessary training can result in misunderstandings, misgendering, and insensitive or inappropriate interactions that undermine trust and rapport between patients and health care professionals [31]. To address these challenges, it is essential to explore and develop tailored educational resources and interventions that provide health care professionals with the knowledge and skills needed to interact effectively with TNBI individuals [32]. Promoting equitable health care access for all individuals requires innovative solutions that empower health care professionals to provide supportive and inclusive care to TNBI patients [32-36]. Incorporating information and communication technology (ICT) into training and education has proven highly beneficial, as it enhances learning opportunities, improves communication, and increases accessibility to educational resources [37]. While there are other strategies for training and education, ICT offers unique advantages such as personalized learning, interactive content, and the ability to reach a wider audience [38]. These benefits make ICT a more effective approach for improving various aspects of education and training. ICT plays a transforming role in health education by providing innovative and accessible solutions to train health care professionals effectively. It also enhances learning flexibility, promotes collaborative opportunities, and ensures scalability to meet diverse educational needs in health care settings [39].

In health care, ICT can be leveraged through digital tools to support training by providing diverse and interactive educational resources, facilitating remote learning, and enabling real-time access to up-to-date medical information and best practices. The World Health Organization defines eHealth as the “cost-effective and secure use of information and communications technologies in support of health and health-related fields, including health-care services, health surveillance, health literature, and health education, knowledge and research” [40]. In this study, eHealth tools refer specifically to the digital health tools (eg, mobile apps and web-based platforms) used by health care professionals for health education purposes. By fostering inclusive practices, these tools can enhance patient trust, reduce discrimination, and ultimately lead to better health outcomes for these populations. Given the limited research on this topic for health care professionals [41], understanding health care professionals’ needs, challenges, and preferences is vital for developing effective, targeted educational resources that promote more inclusive and effective care for TNBI individuals.

### Aim of the Study

This study aimed to explore health care professionals’ perspectives on education and awareness of health issues related to TNBI individuals. Specifically, it aimed to identify their needs, challenges, and preferences in accessing and using digital educational resources to enhance their knowledge and competence in providing inclusive and effective care for this population.

## Methods

### Study Design

A qualitative research approach was chosen to investigate health care professionals’ perspectives on education, awareness, and preferences for digital educational resources to support TNBI care. This method aligns with the study’s aim by comprehensively understanding their experiences, needs, and challenges in accessing and using educational resources. To further enrich this exploration, a sociotechnical framework [42] was applied, as it provides a structured perspective to examine how social, cultural, and technological factors intersect and influence the delivery of inclusive and effective care for TNBI populations. This framework has been applied to examine how health care professionals engage with TNBI individuals and the potential of eHealth educational tools to enhance these interactions. The strengths of qualitative research lie in the ability to gain profound insights into a problem or necessity by directly engaging with individuals and their contexts where the issue arises [43].

### Study Setting and Participants

In total, 15 health care professionals with various health care backgrounds were recruited to participate in this study. The data were collected in Stockholm County, Sweden. While most participant interviews were held via Zoom (Zoom Video Communications), 2 were conducted in person at the participants’ workplaces. The participants were selected through convenience sampling, primarily via social media platforms. The inclusion criteria required participants to be health care professionals with experience in using digital tools in their practice, aged ≥18 years, and proficient in written and spoken English. To capture diverse perspectives, we included professionals across 9 disciplines (physiotherapy, dentistry, pediatrics, general practice, general surgery, gynecology, oncology, psychology, and cosmetic surgery), representing a broad spectrum of patient care. Recruiting participants from different disciplines and countries with varying acceptance, health care system diversity, legal recognition, and social and cultural attitudes toward TNBI individuals enabled us to gather varied insights on the challenges that health care professionals face in interacting with TNBI individuals. Each group of health care professionals in this study plays a crucial role in different aspects of health care, from initial assessments and referrals to specialized care, ongoing support, and mental health services. The inclusion of these varied perspectives was essential for capturing the complexity of care required for TNBI individuals. However, the diversity in the respondent pool also presents challenges, as it can make it more difficult to maintain focused discussions and reach consensus. The exclusion criteria were non–English-speaking health care professionals and individuals without any health care background. These criteria ensured that participants had relevant and thorough experiences, insights, and recommendations related to the research topic, thereby preserving the quality and validity of the study’s findings.

In qualitative research, the number of participants is typically determined by reaching data saturation, meaning that further data collection does not yield new insights [44]. In this study, participants were interviewed until no new insights were generated from the interviews. The participants’ characteristics are presented in Table 1. The participants had a mean age of 40 (SD 7.3) years and worked in various fields within the health care sector. The study included 4 participants from Sweden, 5 participants from India, and 3 participants each from the United States and the United Kingdom. The variation in participant numbers across countries was due to the use of convenience sampling and the differing availability of health care professionals in each discipline and location. Among the 15 participants, 8 (53%) were female, 6 (40%) were male, and 1 (6%) participant did not disclose their sex. In total, 6 (40%) out of 15 participants had no prior experience working with TNBI individuals.

**Table 1:** Participants’ characteristics.

Participant ID	Age range (y)	Geographic location	Occupation	Experience in health care (y)	Experience working with TNBI^a^ individuals
Participant 1	35-45	Sweden	Physiotherapist	5-10	No
Participant 2	30-40	Sweden	Dentist	1-5	No
Participant 3	30-40	Sweden	Pediatrician	5-10	No
Participant 4	30-40	Sweden	Dentist	1-5	Yes
Participant 5	30-40	India	General physician	5-10	Yes
Participant 6	40-50	India	General surgeon	10-15	Yes
Participant 7	50-60	India	Gynecologist	15-20	Yes
Participant 8	50-60	India	Oncologist	1-5	Yes
Participant 9	30-40	India	General practitioner	5-10	No
Participant 10	40-50	United Kingdom	General practitioner	5-10	Yes
Participant 11	30-40	United Kingdom	General surgeon	1-5	No
Participant 12	25-35	United Kingdom	Surgical intern	1-5	No
Participant 13	30-40	United States	Psychologist	5-10	Yes
Participant 14	35-45	United States	General practitioner	5-10	Yes
Participant 15	40-50	United States	Cosmetic surgeon	10-15	Yes

^a^TNBI: transgender, nonbinary, and intersex.

The selected professions were included because they are likely to engage TNBI individuals in their practice. This diversity allows us to capture a wide range of insights and experiences, which are crucial for understanding the multifaceted needs of these patients. Although 6 (40%) out of 15 participants had no prior experience working with TNBI individuals, their contributions were insightful and added significant value to the findings. By including a variety of roles, we aimed to identify common themes and differences across different medical specialties, which can inform more inclusive health care practices.

### Data Collection

#### Overview

Data were collected through semistructured interviews. To formulate our interview script, we followed a systematic approach grounded in the sociotechnical framework by Sittig and Singh [42]. This framework ensures that we comprehensively address the intersection of social, cultural, and technical factors and their impact on providing inclusive and effective care for the TNBI population.

#### Development of the Interview Schedule

The interview schedule (Multimedia Appendix 1) was developed based on the 8 key dimensions of the sociotechnical framework: hardware and software; clinical content; human-computer interface; people; workflow and communication; internal policies, procedures, and culture; external rules and regulations; and measurement and monitoring [42]. We mapped the objectives of our study to the relevant dimensions of the framework to ensure comprehensive coverage: understanding current interactions and challenges (people and workflow and communication), identifying gaps in resources and training (internal policies, procedures, and culture and clinical content), exploring the potential of eHealth tools (hardware and software and human-computer interface), ensuring usability and integration (human-computer interface and communication), considering regulatory and organizational factors (external rules and regulations and internal policies, procedures, and culture), and evaluating effectiveness and feedback mechanisms (measurement and monitoring). We then continued with developing specific questions. For each dimension, we developed specific questions that align with our research objectives. The final interview script was designed to comprehensively address all aspects of the sociotechnical framework, ensuring the collection of detailed data on health care professionals’ perspectives regarding education and awareness of health issues related to TNBI individuals and the potential of digital educational resources to enhance their care delivery. The interview guide was iteratively refined during early interviews to ensure its validity and relevance to the study’s aim.

By using semistructured interviews, we could get a deeper understanding of what the participants had experienced during their clinical practice and their thoughts and feelings related to the need for digital educational resources. Semistructured interviews also allowed for follow-up questions to further deepen the understanding of their thoughts. Each interview lasted between 40 and 60 minutes. All the interviews were conducted in English.

### Data Analysis

**Figure 1:**
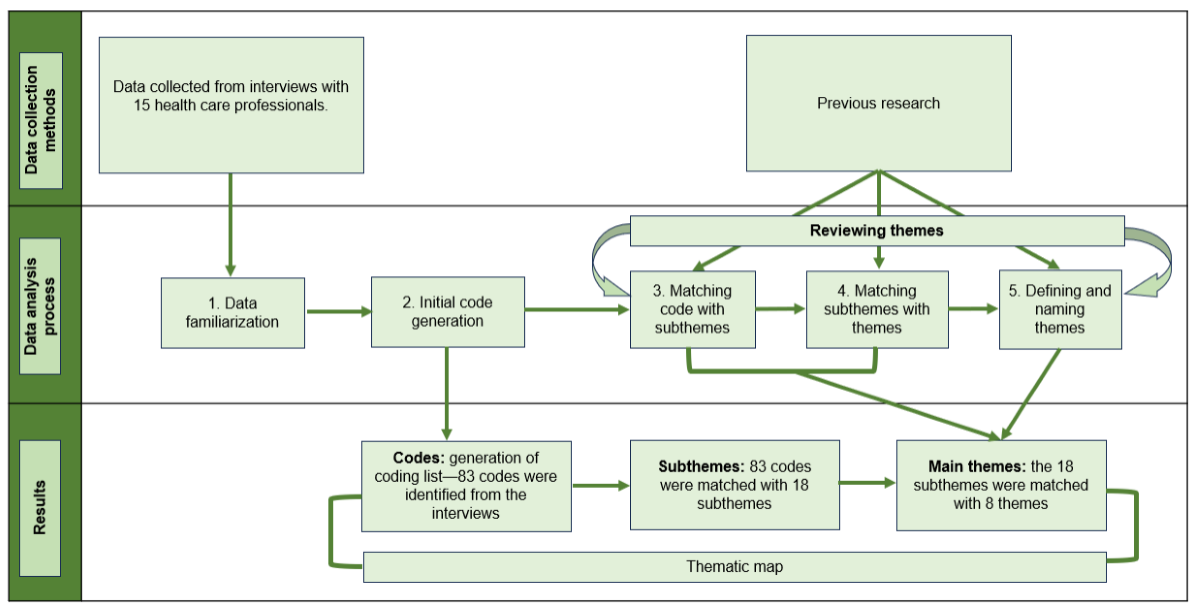
Data analysis process.

The interviews were audio recorded, transcribed, and analyzed using thematic analysis according to the guidelines by Braun and Clarke [45]. The audio recordings were transcribed verbatim using Microsoft Word and then reviewed for accuracy. The transcribed data were first thoroughly reviewed to gain a comprehensive understanding of the data. The next phase involved coding the text where codes are segments of the text that are linked by content and context, allowing a deeper exploration of the underlying themes and concepts. Descriptive coding was used to capture and summarize the main topics of the text. To build on this, pattern coding was applied to condense meaning units into overarching patterns, grouping the initial codes into broader themes. This approach facilitated the identification and understanding of larger patterns and relationships within the data [46]. SK conducted the initial coding by identifying meaning units, condensing them, and assigning relevant codes. Both authors reviewed the initial codes and grouped them into clusters reflecting emerging subthemes and then examined the relationships between the subthemes. They identified patterns and combined related subthemes into broader themes. The data analysis process was an iterative process in which discrepancies were resolved through several meetings and discussions. The authors analyzed the collected data and continuously revisited and refined the codes, subthemes, and themes as needed to ensure a comprehensive understanding of the data. The data analysis process is presented in Figure 1.

### Ethical Considerations

This research was conducted in Sweden. According to the Swedish Ethical Review Act, this study does not require ethics approval as it does not handle sensitive personal information (as defined by the European General Data Protection Regulation). However, ethical requirements still apply. Participants were recruited from Sweden, India, the United States, and the United Kingdom. No sensitive personal information (eg, health status, political opinion, or racial or ethnic background) was collected. Prospective study participants were provided with comprehensive information regarding the study’s objectives, methodologies, potential risks and benefits, and their right to withdraw at any time. This information was conveyed through both a written consent form, which participants were required to sign, as well as verbal explanations provided by the first author. To maintain confidentiality and privacy, the collected data were anonymized. In addition, any personal or sensitive information shared by participants was excluded from the study. The participants were guaranteed confidentiality and informed about how their data would be handled. Although the study was conducted in Sweden, ethical principles such as confidentiality and respect for participants’ autonomy were upheld in line with international research standards, including those applicable in the United States (eg, Common Rule) [47], the United Kingdom (eg, Health Research Authority guidelines) [48], and India (eg, Indian Council of Medical Research guidelines) [49]. These measures ensured the ethical integrity of the research across all participant demographics.

## Results

### Overview

The analysis of the interviews resulted in 8 themes: gender diversity awareness, inclusive communication and understanding of the needs of TNBI individuals, societal structural challenges, regulatory gaps in training and support infrastructure, education and training needs for health care professionals on TNBI care, educational resources and training tools for TNBI care, challenges and design considerations for eHealth tools integration, and evaluating eHealth impact. In addition to the 8 themes, 18 subthemes and 83 codes were formulated. An overview of the sociotechnical aspects, themes, and subthemes is presented in Table 2.

In this study, the participants predominantly used the term *transgender* as an umbrella term to refer broadly to TNBI individuals.

**Table 2:** An overview of the subthemes and themes.

Sociotechnical aspects	Subthemes	Themes
People	Limited understanding of gender diversity	Gender diversity awareness
Workflow and communication	Acknowledgment of communication barriers and the need for inclusive language Lack of understanding of TNBI^a^ individuals’ needs	Inclusive communication and understanding of the needs of TNBI individuals
Internal organizational policies, procedures, and culture	Suppression of identity due to societal stigma, cultural norms, and societal pressures Vulnerability arising from societal and political oppression Limited research on TNBI health issues	Societal and structural challenges
External rules and regulations and pressures	Lack of awareness among health care professionals and inadequate mental health support Gaps in specialized training and guidelines for gender-affirming care Weakness in health care infrastructure for TNBI individuals	Regulatory gaps in training and support infrastructure
Clinical content	Inadequate training in cultural competency regarding gender diversity Importance of education on TNBI health issues for health care professionals Need for tailored resources and training modules designed for mental health professionals	Education and training needs for health care professionals on TNBI care
Hardware and software	Interactive case studies and peer support forums Comprehensive training modules workshops and web-based courses Resources about gender-affirming therapy, trauma, and intersectionality	Educational resources and training tools for TNBI care
Human-computer interface	Challenges in integrating eHealth tools into regular health care practice, including time constraints and cultural change Emphasis on user-friendly design, accessibility features, and data security in health care tools	Challenges and design considerations for eHealth tools integration
System measurement and monitoring	Expectations of improved patient–health care professional relationships using eHealth tools	Evaluating eHealth impact

^a^TNBI: transgender, nonbinary, and intersex.

### Sociotechnical Aspect: People (Theme 1: Gender Diversity Awareness, Subtheme: Limited Understanding of Gender Diversity)

**Figure 2:**
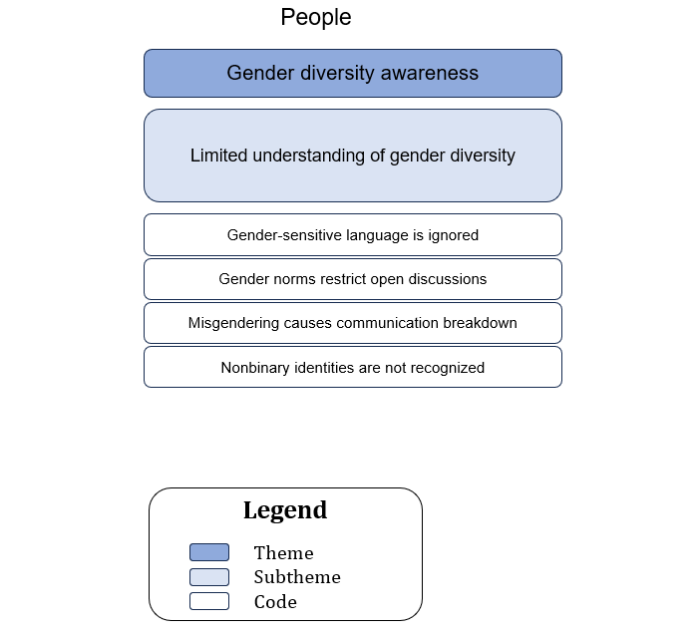
The relationship between the codes and subthemes for theme 1—gender diversity awareness.

A hierarchical structure with the themes at the top, subthemes in the middle, and the corresponding codes at the base is illustrated in Figure 2.

A limited understanding of gender diversity may result in inadequate screening and assessment practices for TNBI patients’ health care needs. Health care professionals discussed misunderstanding the unique health risks and concerns facing TNBI individuals, leading to delays in diagnosis, inappropriate treatment recommendations, or suboptimal care outcomes:

When I worked as an intern, we usually strengthened the biological sex or gender, ignoring the psychological stuff, because most of us in our education, are taught to learn the difference between male and female.

Participant 10

Some participants expressed that a limited understanding of gender diversity could create institutional barriers to accessing gender-affirming care, further exacerbating disparities in health care access and outcomes for TNBI individuals:

From my clinical work, outdated regulations often obstruct the care of transgender patients, a sign of the healthcare system’s failure to fully grasp the nuances of gender diversity.

Participant 11

In my clinical experience, I’ve observed bureaucratic hurdles, such as outdated policies and procedures that fail to accommodate the unique needs of transgender individuals. These stemmed from a limited understanding of gender diversity.

Participant 6

### Sociotechnical Aspect: Workflow and Communication (Theme 2: Inclusive Communication and Understanding the Needs of TNBI Individuals)

**Figure 3:**
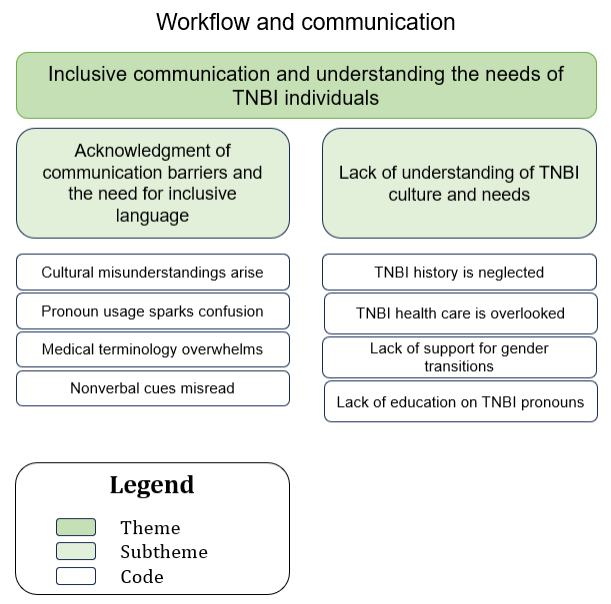
The relationship between the codes and subthemes for theme 2—inclusive communication and understanding the needs of transgender, nonbinary, and intersex (TNBI) individuals.

A hierarchical structure with the themes at the top, subthemes in the middle, and the corresponding codes at the base is illustrated in Figure 3.

#### Acknowledgment of Communication Barriers and the Need for Inclusive Language

Participants felt an existing lack of awareness about TNBI health issues, including appropriate language and communication strategies. They also stated that without adequate education on TNBI terminology and cultural competency, they may unintentionally use insensitive or outdated language, leading to misunderstandings and discomfort for TNBI patients:

I used inappropriate language for communication with transgender patients and they felt bad for that and some of them were even frustrated for not having adequate knowledge of terms that should be used.

Participant 7

I’ve unintentionally used terms that were not affirming, which caused discomfort for my transgender patients, and this has shown me the urgent need for proper education on inclusive language.

Participant 5

Most of the participants presented fear of inadvertently misgendering them. This fear of causing harm or disrespect can lead to hesitation or avoidance of discussions related to gender identity, which can hinder effective communication and rapport building with TNBI patients:

Sometimes I did avoid discussions regarding gender identity, as I was not sure about it, due to which rapport with the patients was not constructive.

Participant 4

Some of the participants mentioned that there are limited resources and guidelines available to health care professionals on best practices for communication with TNBI patients. In the absence of clear guidance, they may struggle to navigate conversations about gender identity and may rely on personal assumptions or biases, which can contribute to communication barriers and misunderstandings:

There were moments when my judgment was clouded by my assumptions leading to uncomfortable situations. It is a mistake I have learned from.

Participant 15

Struggled a lot and also felt embarrassed due to my personal assumptions that led to misunderstandings in a peculiar situation and never did it again.

Participant 6

#### Lack of Understanding of TNBI Culture and Needs

Participants also expressed that they struggled to establish trusting relationships due to a lack of understanding regarding their cultural identities and needs. Therefore, they observed a hindrance in disclosing patient’s gender identity, expressing their health care concerns, and seeking support for their health and well-being:

In fact, I have no experience of working with transgender, hence building rapport with these patients is tough for me as I don’t have a proper idea regarding their needs.

Participant 2

### Sociotechnical Aspect: Internal Organizational Policies, Procedures, and Culture (Theme 3: Societal and Structural Challenges)

**Figure 4:**
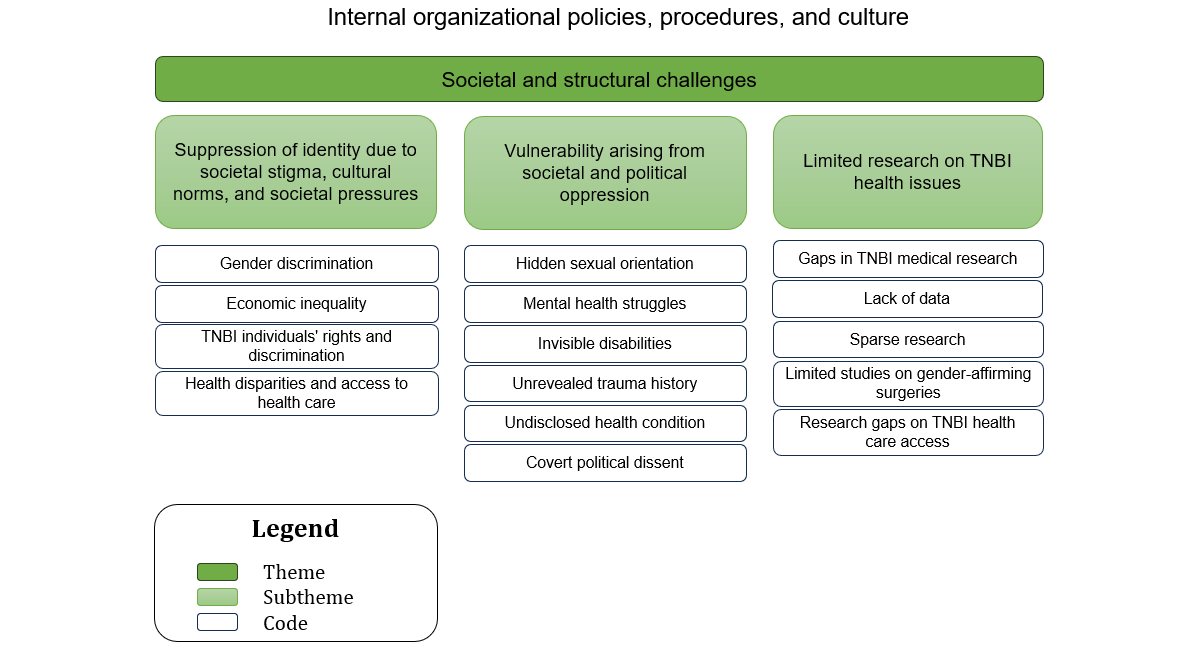
The relationship between the codes and subthemes for theme 3—societal and structural challenges. TNBI: transgender, nonbinary, and intersex.

A hierarchical structure with the themes at the top, subthemes in the middle, and the corresponding codes at the base is illustrated in Figure 4.

#### Suppression of Identity Due to Societal Stigma, Cultural Norms, and Societal Pressures

Participants highlighted that experiences of discrimination, prejudice, and social rejection increased the vulnerability of TNBI individuals to mental health conditions, such as depression, anxiety, and posttraumatic stress disorder. They also believe that internalizing negative stereotypes and beliefs about their identity can further exacerbate mental health issues, causing feelings of shame, self-doubt, and identity concealment:

During my experiences in clinical practice, I have encountered transgender patients who have expressed fear, shame, and hesitation in disclosing their gender identity to healthcare providers.

Participant 10

One of the participants said that traumatic experiences such as hate crimes, physical violence, or verbal abuse based on gender identity can lead to symptoms of posttraumatic stress disorder, including intrusive thoughts, hypervigilance, and avoidance behaviors:

They cannot say how they recognize themselves as transgender or non-binary in the workplace or even to their families. It’s a kind of a secret for them. So, they are emotionally vulnerable and sensitive at the same time.

Participant 13

Many transgender individuals feel they must conceal their identities both professionally and personally, which places them under immense emotional strain and leaves them feeling isolated.

Participant 2

In addition to that, they also focused on the barriers to accessing health care faced by TNBI individuals because of societal stigma. Participants also revealed that past negative experiences or stories of discrimination within health care settings may lead to mistrust and reluctance to seek medical care.

Another point they mentioned is that the fear of being misgendered, invalidated, or subjected to invasive questioning can create significant barriers to accessing necessary health care services. In addition to that, internalized stigma and shame may also contribute to reluctance to seek health care services, as TNBI individuals may feel unworthy of receiving care or fear being perceived as *different*:

The first I think is the culture because trans people and also non-binary people, have their own culture, different than the majority culture and we, health care professionals didn’t know that.

Participant 5

Some of the participants mentioned that using correct pronouns and respecting chosen names can help mitigate the effects of societal stigma and foster a sense of validation and belonging for TNBI patients:

It reminds me of a transgender patient, who did not behave like the gender that person looked like and that causes some confusion. So, it’s necessary to ask them first how you recognize yourself and respect their social gender identification.

Participant 5

Gender identity is personal, and as clinicians, we should always lead with questions, not assumptions to ensure we are providing care that respects who they are.

Participant 8

#### Vulnerability Arising From Societal and Political Oppression

For TNBI individuals, intersectionality exacerbates vulnerability, particularly for those who belong to marginalized racial, ethnic, or socioeconomic groups. This means that TNBI individuals may face compounded discrimination and barriers to health care access due to overlapping forms of oppression:

I have seen many cases, who faced added discrimination to healthcare access due to political oppression...I believe that it’s crucial to understand how multiple forms of discrimination intersect for transgender people, making access to healthcare even more challenging.

Participant 6

Political and social discrimination compounds the barriers transgender individuals face in accessing care, highlighting the need to address overlapping layers of bias in healthcare systems.

Participant 11

#### Limited Research on TNBI Health Issues

Participants said that they rely on evidence-based practices to guide their clinical decision-making and provide quality care to patients. The limited research on TNBI health issues means that there may be a lack of robust evidence to inform best practices in the diagnosis, treatment, and management of health conditions:

Without sufficient research, we are not only constrained in delivering optimal care, but it is also difficult to fully trust the treatments we prescribe to our patients.

Participant 9

Lack of research not only hampers our ability to deliver effective care but also undermines our confidence in the treatments and interventions we provide [...] As a result, we often find ourselves relying on anecdotal evidence, expert opinions, and extrapolations from related fields to inform our clinical decisions.

Participant 4

They also reported the lack of data on prevalence, risk factors, and outcomes of health conditions within these populations as a hindrance to their ability to assess and address their health care needs accurately. Without this information, it is challenging to determine the scope and magnitude of health disparities and to allocate resources effectively to address them:

Without accurate data, it’s challenging to develop informed strategies for promoting the health and well-being of transgender and non-binary communities.

Participant 3

The absence of prevalence data makes it difficult to gauge the extent of health disparities within transgender and non-binary populations.

Participant 13

In addition, the lack of data on risk factors means that health care professionals may struggle to identify and mitigate factors that contribute to adverse health outcomes among TNBI individuals. Furthermore, the absence of data on outcomes of health conditions within TNBI populations hampers efforts to evaluate the effectiveness of interventions and treatments. Without data on treatment outcomes, health care professionals may be limited in their ability to tailor interventions to the unique needs of TNBI individuals and to optimize their health outcomes:

We struggle to allocate resources effectively and prioritize interventions without a clear understanding of the prevalence and severity of health conditions among transgender and non-binary individuals.

Participant 5

Participants also recognized the importance of investing in research initiatives focused on TNBI health to fill existing knowledge gaps and improve health care delivery:

It’s necessary that we prioritize funding and resources for research aimed at filling existing knowledge gaps, addressing disparities, and promoting health equity among transgender and non-binary populations in transgender and non-binary healthcare.

Participant 4

### Sociotechnical Aspect: External Rules and Regulations and Pressures (Theme 4: Regulatory Gaps in Training and Support Infrastructure)

**Figure 5:**
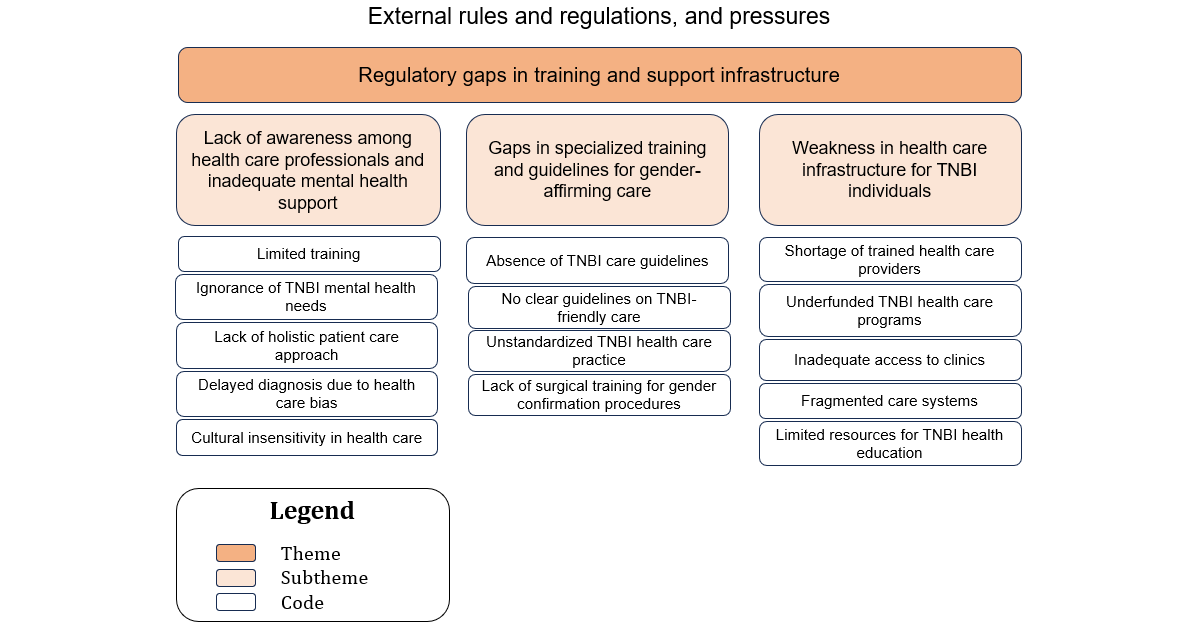
The relationship between the codes and subthemes for theme 4—regulatory gaps in training and support infrastructure. TNBI: transgender, nonbinary, and intersex.

A hierarchical structure with the themes at the top, subthemes in the middle, and the corresponding codes at the base is illustrated in Figure 5.

#### Lack of Awareness Among Health Care Professionals and Inadequate Mental Health Support

Significant gaps discussed by the participants include a lack of awareness and understanding of TNBI health issues. They also expressed feelings of being frustrated by the systemic gaps in education and training on TNBI health issues within their profession. They were also concerned about the limited availability of culturally competent and affirming mental health services for TNBI patients, recognizing the detrimental impact of untreated mental health conditions on their overall well-being:

I feel ill-equipped to address their unique healthcare needs, leading to challenges in providing culturally competent and affirming care.

Participant 3

#### Gaps in Specialized Training and Guidelines for Gender-Affirming Care

The participants’ identification of gaps in specialized training and guidelines for gender-affirming care highlights a critical challenge within health care systems. These gaps stem from limited education and training on transgender health issues and gender-affirming care during both formal education and professional training programs. They also expressed concerns about the lack of comprehensive education and training on transgender health topics throughout their academic and professional journeys:

I feel the lack of preparation and education on transgender health issues is a systemic issue that requires immediate attention and action from healthcare institutions and educational programs.

Participant 3

Many reported minimal exposure to transgender health issues and gender-affirming care protocols during their formal education, which left them feeling ill-prepared to provide culturally competent and affirming care to transgender patients.

In contrast, they expressed that they are encountering inconsistencies in clinical protocols, treatment approaches, and referral criteria for TNBI patients, resulting in variations in care quality and patient experiences due to the lack of standardized guidelines within health care institutions and disciplines.

Even though some of the participants were interested in specializing in transgender health, they said that they were facing challenges in accessing advanced training opportunities to develop expertise in this area, leading to a scarcity of trained specialists within the health care workforce:

Even with a strong interest in transgender healthcare, I’m struggling to access the specialized training necessary to develop my skills in this field.

Participant 12

Despite my interest in specializing in transgender health, I am encountering challenges in accessing advanced training opportunities.

Participant 13

#### Weaknesses in Health Care Infrastructure for TNBI Individuals

The participants also stated that they observed a lack of specialized health care facilities equipped to provide gender-affirming care for TNBI individuals. In addition, they also reported the absence of dedicated clinics or centers specializing in transgender health, which limits access to competent care and contributes to disparities in health outcomes:

There’s a noticeable absence of dedicated clinics specializing in transgender health within our healthcare system, due to which patients are facing difficulties in receiving the affirming care they need.

Participant 6

The scarcity of transgender-focused healthcare facilities means many patients struggle to find affirming care, which compromises their overall well-being.

Participant 4

They have also mentioned the challenges they were facing in accessing gender-affirming treatments, such as hormone therapy and gender-affirming surgeries, for TNBI individuals, including a shortage of trained health care providers and long waiting times for appointments. Apart from the aforementioned challenges, they also recognized the need for expanded access to mental health support services for TNBI individuals:

The limited availability of trained providers is impeding my ability to provide timely and comprehensive gender-affirming care.

Participant 4

The shortage of providers skilled in transgender care significantly delays access to the affirming treatments many patients require.

Participant 8

For transgender individuals, mental health support is essential for comprehensive care, yet it remains frustratingly inadequate in many healthcare settings.

Participant 9

Access to mental health support services is crucial for the holistic well-being of transgender individuals, yet it remains limited in many healthcare settings.

Participant 6

### Sociotechnical Aspect: Clinical Content (Theme 5: Education and Training Needs for Health Care Professionals on TNBI Care)

**Figure 6:**
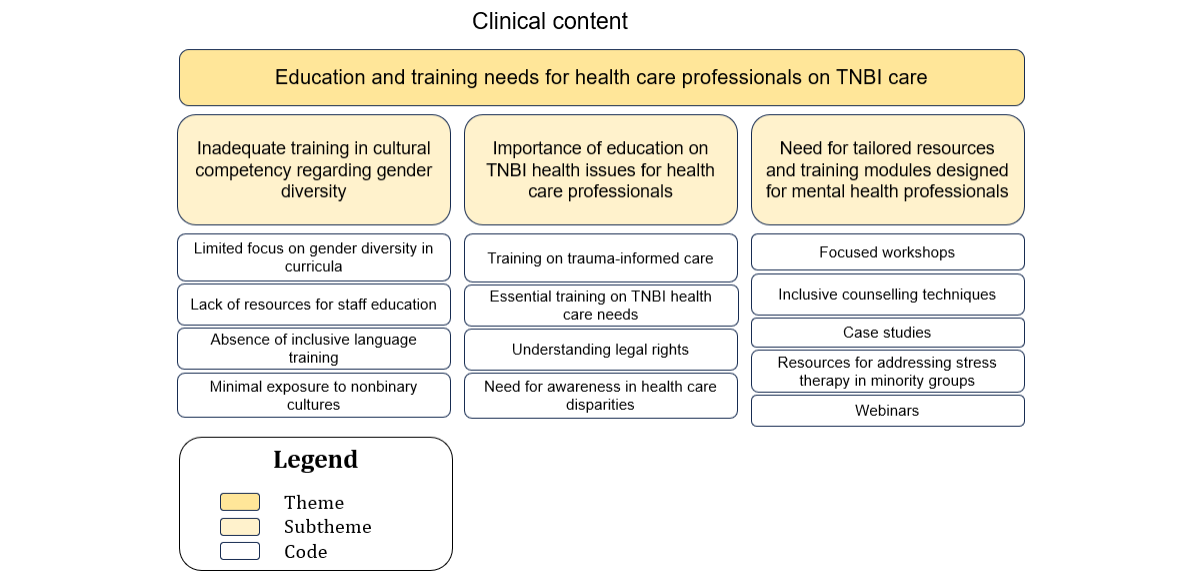
The relationship between the codes and subthemes for theme 5—education and training needs for health care professionals on transgender, nonbinary, and intersex (TNBI) care.

A hierarchical structure with the themes at the top, subthemes in the middle, and the corresponding codes at the base is illustrated in Figure 6.

#### Inadequate Training in Cultural Competency Regarding Gender Diversity

Participants reported a lack of comprehensive instructions on gender diversity and cultural competency during their formal education and professional training. Many indicated that TNBI health topics were either inadequately covered or entirely omitted from their curriculum. Without proper education and training in this area, health care professionals find themselves inadequately prepared to navigate the complex landscape of gender identity and expression:

Without adequate training in transgender health, many of us enter practice unsure of how to approach the nuanced aspects of gender identity and provide affirming care to all patients.

Participant 9

The limited coverage of transgender health topics in our curriculum left us feeling ill-equipped to navigate the nuances of gender identity and expression in clinical practice.

Participant 4

Our training offered minimal focus on transgender health, leaving us underprepared to address the complexities of gender identity in practice.

Participant 5

Participants also expressed their struggles to provide patient-centered care that respects and affirms the diverse identities and needs of transgender patients, resulting in disparities in health care access, quality, and satisfaction:

I can say that I have experienced difficulties in tailoring care approaches to align with the diverse identities and needs of transgender patients. My attempts to provide affirming care to transgender patients have revealed gaps in my understanding and implementation of patient-centered principles.

Participant 6

#### Importance of Education on TNBI Health Issues for Health Care Professionals

Participants felt that education on TNBI health fosters the creation of inclusive health care environments where all patients feel respected, affirmed, and understood. Health care professionals who received training on transgender health issues are better equipped to provide culturally competent care, use affirming language, and create safe spaces for transgender patients to access health care without fear of discrimination or mistreatment:

I strongly believe education on transgender health is a crucial step towards building a healthcare system that is truly inclusive and affirming of all gender identities.

Participant 5

I assume by investing in education on transgender health, healthcare institutions can promote inclusivity and reduce disparities in healthcare access and outcomes for transgender individuals.

Participant 7

Investing in education about transgender health is a critical step toward fostering inclusivity and addressing disparities in care outcomes.

Participant 9

They also felt that education regarding TNBI health issues empowers them to recognize and address barriers, including delayed diagnosis and inappropriate treatment, ensuring that TNBI individuals receive timely, appropriate, and affirming health care services that meet their unique needs:

By educating ourselves on transgender health issues, we can break down barriers and create more inclusive healthcare environments and we can also work towards reducing disparities in healthcare access and outcomes for transgender individuals.

Participant 10

Through education on transgender health issues, we can eliminate obstacles to care and strive to reduce healthcare disparities for transgender individuals, ensuring better outcomes for all.

Participant 12

Participants were also interested in learning about the specific health care needs of TNBI patients, including hormone therapy, gender-affirming surgeries, preventive care, and mental health support, enabling them to deliver comprehensive and evidence-based care:

At least, I am eager to gain insights into the unique healthcare needs of transgender patients, including understanding nuances of hormone therapy and this interest in learning about the healthcare needs of transgender patients reflects our commitment to providing inclusive and affirming care within our practice.

Participant 4

#### Need for Tailored Resources and Training Modules Specifically Designed for Mental Health Professionals

The need for specialized training for mental health professionals to understand the unique mental health challenges faced by TNBI individuals has been observed by some participants, as these individuals are at increased risk of mental health disorders such as depression, anxiety, suicidality, and gender dysphoria due to societal stigma, discrimination, and identity-related stressors.

One of the participants, who was a mental health professional, was also expecting training in gender-affirming care principles to provide affirming and culturally competent mental health services to transgender and gender-diverse clients. The participant feels that tailored resources and training modules equip mental health professionals with strategies for creating affirming therapeutic environments and implementing gender-affirming interventions:

As per my observation, through education and training, mental health professionals can gain the knowledge and skills needed to provide competent and compassionate care to transgender and gender-diverse clients.

Participant 13

### Sociotechnical Aspect: Hardware and Software (Theme 6: Educational Resources and Training Tools for TNBI Care)

**Figure 7:**
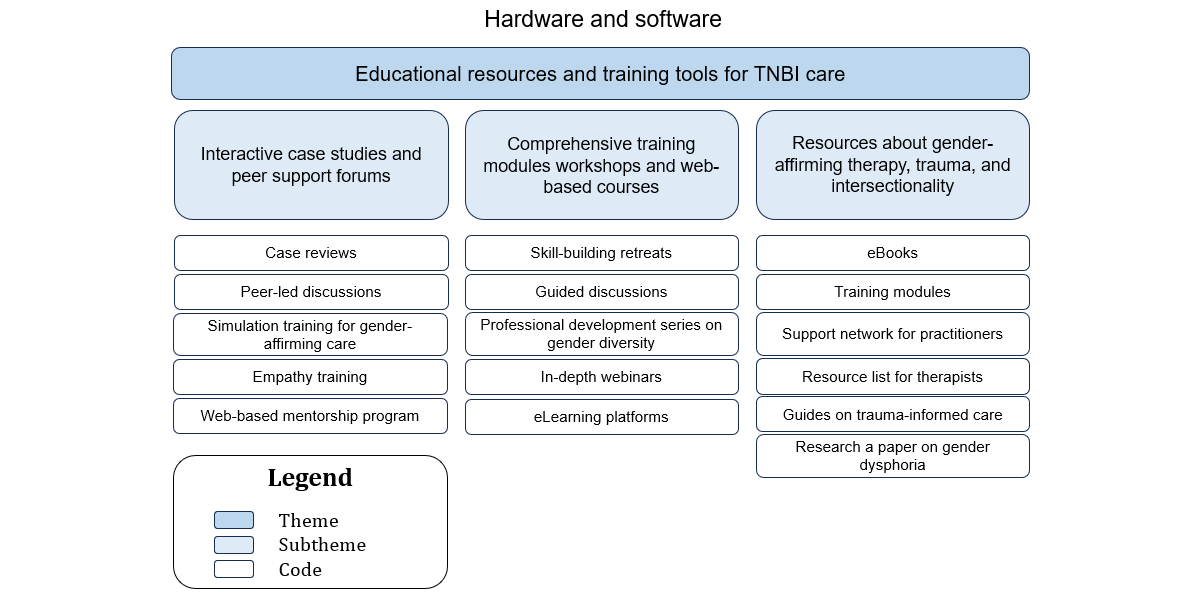
The relationship between the codes and subthemes for theme 6—educational resources and training tools for transgender, nonbinary, and intersex (TNBI) care.

A hierarchical structure with the themes at the top, subthemes in the middle, and the corresponding codes at the base is illustrated in Figure 7.

#### Interactive Case Studies and Peer Support Forums

The participants generally perceived interactive case studies and peer support forums as valuable educational resources that promote active learning, cultural competency, professional networking, and skill development in transgender health.

Some of them felt that interactive case studies allow them to engage actively in the learning process by analyzing real-life scenarios, making decisions, and receiving immediate feedback:

My personal preference is to have interactive case studies, as they enable active participation and engagement in the learning process.

Participant 7

Others were interested in peer support forums that provide opportunities to learn from each other’s experiences, perspectives, and insights, fostering a collaborative learning environment. They strongly believed that peer support forums facilitate knowledge sharing, discussion of clinical challenges, and exchange of best practices among peers, leading to enhanced learning outcomes and professional development.

They also felt that they could access these resources at their convenience to refresh their knowledge, stay updated on emerging practices, and enhance their clinical competencies in transgender health:

I agree that these resources encourage reflection, critical thinking, and dialogue among health care professionals, promoting continuous improvement in transgender healthcare delivery.

Participant 3

#### Comprehensive Training Modules, Workshops, and Web-Based Courses Focusing on Gender Diversity

To have an in-depth understanding of gender diversity, including the spectrum of gender identities and expressions, health care professionals were in favor of comprehensive training modules, workshops, and web-based courses.

They expect the training modules, workshops, and web-based courses to facilitate interdisciplinary collaboration among themselves from different specialties and disciplines, leading them to work collaboratively as part of multidisciplinary care teams to address the complex health care needs of transgender and gender-diverse patients, promoting coordinated and holistic care approaches:

The opportunity to exchange knowledge and best practices among health care professionals is vital for advancing our shared understanding of transgender health issues.

Participant 11

Some participants felt that training modules, workshops, and web-based courses focusing on gender diversity contribute to advancing health equity and social justice for transgender and nonbinary gender-diverse individuals. On the contrary, one of the participants expressed concern regarding time management:

In my opinion, it’s essential to tackle issues regarding time management and resource allocation to ensure health care professionals can actively participate in gender diversity training and play their part in promoting health equity and social justice.

Participant 2

#### Resources About Gender-Affirming Therapy, Trauma, and Intersectionality

Health care professionals are expected to have resources that cover principles and practices of gender-affirming care, including approaches to hormone therapy, gender-affirming surgeries, and psychotherapy, enabling them to support transgender patients in aligning their bodies with their gender identities:

I expect to have access to resources on gender-affirming care will equip us with the knowledge and skills needed to navigate complex healthcare decisions and provide holistic support to transgender patients throughout their transition journey.

Participant 3

In addition, they were willing to have and learn about trauma-sensitive approaches to care delivery, recognizing the impact of past traumas on patients’ mental health and well-being and creating safe and supportive environments for survivors of trauma to access health care services:

I am open to learning and implementing trauma-sensitive approaches into our practice, as I believe they are essential for providing patient-centered and holistic care to individuals affected by trauma.

Participant 13

As health care professionals, we recognize the importance of ongoing education and training in trauma-sensitive care to ensure that we can effectively meet the needs of patients who have experienced trauma.

Participant 10

Health care professionals were interested in gaining an understanding of how multiple intersecting identities influence individuals’ experiences of discrimination, access to health care, and health outcomes, enabling them to provide more nuanced and inclusive care to diverse transgender communities:

My priority is having education about intersectionality to work towards creating more equitable and inclusive healthcare systems that address the complex needs of all patients.

Participant 13

### Sociotechnical Aspect: Human-Computer Interface (Theme 7: Challenges and Design Considerations for eHealth Tools Integration)

**Figure 8:**
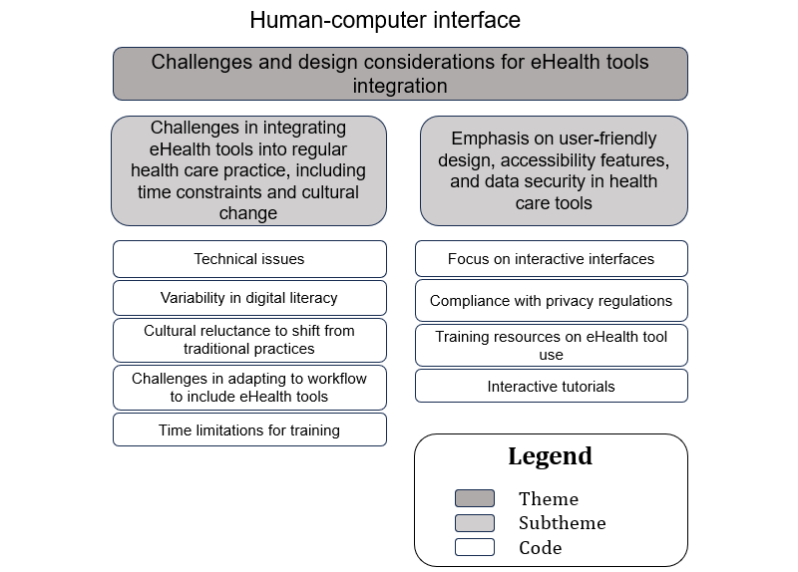
The relationship between the codes and subthemes for theme 7—challenges and design considerations for eHealth tools integration.

A hierarchical structure with the themes at the top, subthemes in the middle, and the corresponding codes at the base is illustrated in Figure 8.

#### Challenges in Integrating eHealth Tools Into Regular Health Care Practice, Including Time Constraints and Cultural Change

Several challenges were recognized in integrating eHealth tools into regular health care practice, including time constraints and cultural change. Participants said that they often face time constraints due to busy schedules, heavy workloads, and competing priorities in clinical practice.

They recognized that integrating eHealth tools could require additional time for training, learning new technologies, documentation, and troubleshooting technical issues. This demand may strain already limited time resources and disrupt workflow efficiency. Participants acknowledged that integrating eHealth tools requires a cultural shift in attitudes, behaviors, and practices within health care organizations and among health care professionals:

With our heavy workloads, it can be challenging to dedicate time to training and troubleshooting the technical issues that arise with eHealth tools.

Participant 14

Require additional time for documentation and adapting to new workflows, which can strain our already limited time resources.

Participant 7

They recognized that resistance to change, skepticism about technology, and concerns about the impact on traditional care delivery models may impede the adoption and acceptance of eHealth tools, necessitating cultural change initiatives, leadership support, and stakeholder engagement to foster a culture of innovation and digital transformation:

In the place I work, to overcome resistance to change, proactive efforts are required to educate, train, and support healthcare staff in adopting new technologies and workflows.

Participant 7

One participant anticipated that integrating eHealth tools into regular health care practice may disrupt existing workflows and processes, leading to initial challenges in adaptation and implementation. They expressed concerns about potential workflow inefficiencies, disruptions in patient flow, and coordination issues among health care team members, particularly during the transition phase, when adapting to new technologies and integrating them into clinical routines:

It would be difficult for most of us during the transition phase, as there may be a need for additional training, support, and resources to help us navigate the changes and overcome implementation barriers.

Participant 13

Participants expressed that they even encounter technical and logistical barriers, such as inadequate infrastructure, limited access to technology, interoperability challenges, and data security concerns, which hinder the seamless integration of eHealth tools into regular health care practice. They recognized the need for investment in IT infrastructure, resources for training and support, and adherence to regulatory requirements and standards to address these barriers effectively and ensure the successful implementation and use of eHealth tools:

Inadequate infrastructure and limited access to technology might hinder the seamless integration of eHealth tools into our practice.

Participant 3

They recognized that patients may have varying levels of comfort, literacy, and access to technology, which can influence their willingness and ability to engage with eHealth tools. Health care professionals emphasized the need for patient education, support, and empowerment to promote successful integration and use of eHealth tools in regular health care practice:

As health care professionals, we play a critical role in facilitating patient engagement and empowerment in the use of eHealth tools by providing guidance, encouragement, and ongoing support.

Participant 15

#### Emphasis on User-Friendly Design, Accessibility Features, and Data Security in eHealth Tools

Health care professionals prioritized user-friendly design in eHealth tools to ensure ease of use, intuitive navigation, and efficient workflow integration. They recognize that user-friendly interfaces enhance usability, minimize user errors, and promote acceptance and adoption among health care professionals, ultimately improving efficiency and productivity in clinical practice.

Health care professionals prioritized data security and privacy in eHealth tools to protect sensitive patient information, maintain confidentiality, and comply with regulatory requirements:

I prefer the user-friendly design of eHealth tools to ensure that we can easily navigate and utilize these technologies in our daily practice.

Participant 4

Health care professionals recognized that user-friendly design and accessibility features in eHealth tools contribute to patient engagement and empowerment. They believed that tools that are easy to use, accessible across devices, and customizable to individual preferences encourage active participation, self-management, and shared decision-making among patients, leading to improved health outcomes and patient satisfaction. Health care professionals’ confidence and satisfaction with eHealth tools are influenced by the design, usability, and security features of these tools.

They expressed a preference for tools that are intuitive, reliable, and secure, enabling them to focus on patient care rather than technical frustrations or concerns about data integrity, thereby enhancing their overall professional experience and job satisfaction:

I believe that patient-centric design principles, such as intuitive interfaces and clear navigation, are essential for promoting patient autonomy and engagement in their healthcare.

Participant 2

### Sociotechnical Aspect: System Measurement and Monitoring (Theme 8: Evaluating eHealth Impact, Subtheme: Expectations of Improved Patient–Health Care Professional Relationships Using eHealth Tools)

**Figure 9:**
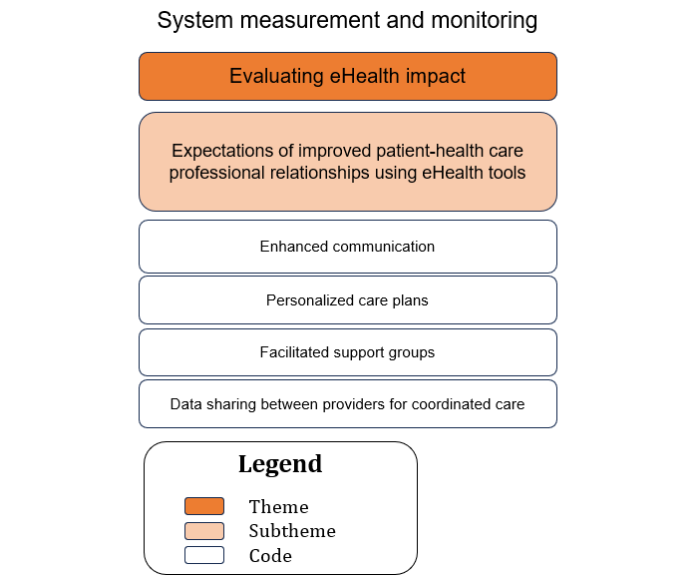
The relationship between the codes and subthemes for theme 8—evaluating eHealth impact.

A hierarchical structure with the themes at the top, subthemes in the middle, and the corresponding codes at the base is illustrated in Figure 9.

Health care professionals anticipated that eHealth tools would enhance communication, enabling more frequent and accessible contact with patients. This, in turn, could strengthen the patient–health care professional relationship by fostering trust, engagement, and continuity of care:

I still work in a healthcare setting, where there is no eHealth tools included in the regular practice. Through the integration of eHealth tools into our practice, we aim to create a healthcare environment that values transparency, accessibility, and patient engagement.

Participant 7

## Discussion

### Principal Findings and Comparison With Prior Work

#### Overview

The overall theme of this study is enhancing health care professionals’ education and awareness of inclusive TNBI care. The analysis of health care professionals’ interviews revealed significant insights into the challenges they face in providing care to TNBI individuals and the gaps in health care systems regarding these individuals’ health. The analysis also highlighted the pivotal role of eHealth educational tools in addressing various challenges faced by health care professionals in providing inclusive and affirming care to transgender and gender-diverse individuals. Through its features and functionalities, an educational tool aims to bridge gaps in knowledge, communication, and access to resources, ultimately enhancing health care delivery and patient outcomes. In total, 8 themes were identified, highlighting the multifaceted nature of TNBI health care and the complex interplay among societal, cultural, regulations, institutional, technical, and individual factors.

Societal stigma and structural challenges emerged as a pervasive issue affecting the health and well-being of TNBI individuals. Participants highlighted the detrimental effects of discrimination, prejudice, and social rejection on mental health, emphasizing the need for culturally competent and affirming care to mitigate these challenges. In countries such as the United Kingdom and the United States, social acceptance and legal protections for TNBI individuals have progressed significantly, with antidiscrimination laws and health care rights actively supporting gender diversity. However, unlike in India, where acceptance remains complex due to strong cultural and religious norms, participants from these Western countries still reported that many patients experience fear, shame, and hesitation when disclosing their gender identity—whether to health care professionals, in the workplace, or even within their families. The fear of being misgendered or invalidated, coupled with past traumas and societal pressures, creates significant barriers to accessing health care services, underscoring the importance of creating safe and supportive environments within health care settings.

Regarding external rules and regulatory gaps, although the United Kingdom and the United States are generally more open in terms of social and cultural norms, the recent sociopolitical shift in both countries has negatively affected TNBI care. For example, the United Kingdom has recently removed gender-affirming care with puberty blockers for those aged <18 years [50]. In the United States, >20 states have aimed to limit or ban access to gender-affirming care, especially for minors. These measures also included restrictions on access to mental health support and educational resources for TNBI individuals. Such policies reflect a growing sociopolitical climate that not only affects the patients directly but also impacts the engagement of health care professionals in providing gender-affirming care. These changes can lead to decreased accessibility to necessary treatments and negatively affect health outcomes for TNBI individuals [51].

Gender diversity awareness, inclusive communication, and understanding the needs of TNBI individuals have revealed a lack of awareness and understanding of TNBI health issues among health care professionals. Communication barriers, limited education on transgender and nonbinary terminology, and inadequate training in cultural competency contribute to misunderstandings and discomfort for these individuals. In addition, the limited understanding of gender diversity and institutional barriers further hinder effective care delivery, highlighting the need for comprehensive education and training on transgender and nonbinary health topics. Participants in this study noted that despite social acceptance, a lack of clear guidance and limited resources leaves them struggling with issues around gender identity. They expressed a fear of misgendering individuals and often avoided discussions on gender identity. Participants mentioned that their training has emphasized focusing on biological sex in treatment, often overlooking the psychological aspects of gender identity.

Various systemic issues, including the lack of awareness among health care professionals, limited mental health support, and gaps in research and specialized training for gender-affirming care, were identified as substantial barriers to providing comprehensive care for TNBI individuals. All participants emphasized the need for increased awareness, research, and resources to address these gaps and promote health equity and social justice for TNBI individuals.

Education and training needs for health care professionals on TNBI care highlighted the importance of comprehensive education on gender diversity and transgender and nonbinary health issues for health care professionals. Participants from all 4 countries expressed a desire for tailored resources and training modules specifically designed to enhance cultural competency and provide gender-affirming care. Moreover, the lack of specialized training and guidelines for gender-affirming care underscores the need for systemic changes within health care institutions to support the professional development of health care professionals in this area.

Educational resources and training tools for TNBI care identified interactive case studies, peer support forums, and comprehensive training modules as valuable tools for promoting active learning and skill development in transgender and nonbinary health. These resources facilitate knowledge sharing, collaboration, and reflection among health care professionals, ultimately enhancing the quality of care for these individuals.

Challenges and design considerations for eHealth tools integration and evaluating eHealth impact presented both opportunities and challenges in health care delivery. While eHealth tools have the potential to streamline communication and improve patient–health care professional relationships, participants identified various barriers, including time constraints, cultural change, and technical issues. Overcoming these barriers requires proactive efforts to address resistance to change, invest in IT infrastructure, and prioritize user-friendly design and data security.

#### Subanalysis of Participants With Experience in Providing Health Care to TNBI Individuals and Those Without Such Experience

Most of the participants without experience in working with TNBI individuals felt ill-equipped to address their unique health care needs. They struggled to build trusting relationships due to a limited understanding of cultural identities and health care requirements, citing that their education focused solely on binary male and female individuals’ perspectives. Participants from Sweden noted that, despite living in an open society, gaps in transgender and nonbinary-specific training persist.

Both groups emphasized that delivering trustworthy care requires sufficient research and evidence-based practice to guide clinical decisions. There was no perceived difference in mental health support for TNBI individuals across Sweden, India, the United States, and the United Kingdom. All participants—regardless of prior experience—reported a lack of comprehensive training and instructions on TNBI health, with a shared belief that education is crucial for fostering an inclusive and affirming health care system. Participants across countries agreed that exchanging knowledge, ongoing training, and access to gender-affirming resources are essential for equipping health care professionals. While many were optimistic about eHealth tools improving communication with patients and continued education, they noted challenges such as additional training requirements, adapting to new workflows, and the importance of user-friendly designs.

The findings of this study align with and extend upon existing research in the field of health care education, particularly regarding the communication needs of health care professionals when interacting with TNBI individuals.

Hughto et al [33] highlighted the importance of addressing societal stigma and vulnerability in health care settings, particularly for TNBI individuals. Similarly, our research underscores the detrimental effects of societal stigma and cultural norms on TNBI mental health and well-being, as well as the barriers to accessing health care services due to fear of discrimination and misgendering.

Furthermore, our study supports previous research conducted by Grant et al [22] on the challenges faced by health care professionals in delivering inclusive and affirming care to transgender patients. Consistent with these findings, our participants expressed concerns about communication barriers, limited understanding of gender diversity, and gaps in specialized training and guidelines for gender-affirming care. These challenges highlight the need for comprehensive education and training initiatives to enhance health care professionals’ cultural competency and sensitivity to transgender and nonbinary health issues.

A scoping review of transgender health training in internal medicine and subspecialty residency programs identified significant gaps in medical education, emphasizing the need for clearly defined objectives to prepare health care professionals for competent and affirming transgender care [52]. Similarly, a systematic review of educational interventions for medical students and residents working with sexual and gender-minority patients demonstrated the effectiveness of structured programs in improving knowledge, attitudes, confidence, and skills, highlighting the importance of implementing comprehensive training to bridge these gaps [53]. Davidge-Pitts et al [54] studied the importance of comprehensive training and educational resources to address the gaps in health care professional’s knowledge and skills related to TNBI health issues. Similarly, our study underscores the significance of tailored educational tools in enhancing health care professional’s understanding and competency in providing gender-affirming care. In addition, the Transgender Education for Affirmative and Competent HIV and Healthcare Program further highlights the impact of structured educational initiatives in fostering gender-affirming knowledge, perceived competency, and inclusive practice behaviors among health care providers [55]. These findings align with our study’s recommendation for interactive, solution-oriented tools to promote ongoing skill development and professional collaboration. In addition, research on barriers to transgender and gender-diverse care highlights several challenges, including the absence of clear guidelines; extended waiting times; a shortage of specialist centers; insufficient training in transgender and gender-diverse health; and technical, cultural, and social obstacles [26]. These findings align with and reinforce the results of our study.

Our study supports the notion that interactive case studies and peer support forums, integrated within an educational tool, promote active learning, cultural competency, and professional networking among health care professionals. These features facilitate ongoing learning and skill development, fostering a collaborative environment conducive to improving TNBI health care delivery. Moreover, the challenges identified in the integration and use of eHealth tools, as discussed in our study, echo the findings of previous research by Light et al [56]. Time constraints, cultural barriers, and technical issues have consistently been recognized as barriers to the adoption of digital health technologies in clinical practice by the participants.

In contrast to earlier studies conducted by Mansh et al [57], which primarily focused on identifying gaps and challenges in TNBI health care education, our study advances the field by proposing a solution-oriented approach. This study provides actionable recommendations for improving TNBI health care education and training initiatives. This shift toward solution-focused research aligns with the broader goal of enhancing health care delivery and patient outcomes through innovative educational interventions. The findings of this study contribute to the growing body of literature on educational interventions for health care professionals and their implications for TNBI health.

### Strengths and Limitations of the Study

The main strength of this study is the use of a qualitative research approach, which allowed for an in-depth exploration of the challenges faced by health care professionals when communicating with TNBI individuals, facilitating a comprehensive understanding of the nuances and complexities surrounding this topic.

In addition, the study included participants from various health care backgrounds and geographic locations, enhancing the richness and diversity of perspectives gathered. This diversity contributed to a more comprehensive analysis of the educational eHealth tools’ requirements and potential impact across different health care contexts. The diverse backgrounds of the participants offered a wide range of experiences and provided a more comprehensive understanding of the issues. While our study provides a broad overview of the needs across different medical professions, we recognize the importance of conducting more in-depth studies for each profession. Future research should focus on the specific needs and challenges faced by each medical specialty when working with TNBI individuals. As qualitative studies do not guarantee the generalizability of the results, we argue that the results of our study are transferable to other contexts.

We believe that the number of interviews conducted with health care professionals was sufficient to achieve data saturation across the overall sample. This indicates that we obtained a sufficient breadth and depth of information to address the research objectives. This ensured that a thorough exploration of the topic was achieved without the need for additional participants [37]. It is also important to note that due to the diversity of countries and health care professions in the sample, combined with the relatively low number of participants from each profession and country, data saturation specific to each subgroup may not have been fully achieved. This limitation could affect the transferability of our findings, particularly for specific roles or cultural contexts. Future research could benefit from a more focused examination of these subgroups to deepen the understanding of context-specific nuances. In addition, conducting additional interviews with health care professionals from settings not represented in this study could enrich findings within the subject.

Another limitation of the study is that the interview guide was not pilot-tested. However, the authors carefully designed the guide and conducted multiple meetings to review and refine it. In addition, during the interviews, we maintained a flexible and adaptive approach, making minor adjustments needed to better capture health care professionals’ experiences.

### Conclusions

The study aimed to explore health care professionals’ perspectives on education, awareness, and preferences for digital educational resources to support TNBI care. The results provided valuable insights into the barriers health care professionals encounter when providing care to TNBI. The study identified key gaps in health care professionals’ understanding of gender diversity, cultural competency, and the need for inclusive communication. In addition, the study emphasized the importance of specialized training and the integration of user-friendly eHealth tools to improve the relationships between health care professionals with TNBI individuals.

eHealth tools play a significant role in enhancing patient–health care professional relationships, improving access to care, and promoting patient engagement in health care. Despite the challenges associated with their integration, health care professionals acknowledged their potential to facilitate more efficient, patient-centered care delivery.

Addressing the identified barriers and challenges through targeted interventions, such as providing training and support for health care professionals, investing in user-friendly design and data security, and promoting cultural competence in providing health care for TNBI individuals, is essential.

In conclusion, this study contributes to the growing literature on eHealth interventions in TNBI health care and sets the stage for future research and practice initiatives aimed at leveraging technology to improve health outcomes and reduce health disparities for these individuals.

## Appendix

Multimedia Appendix 1 Interview guide.

## Abbreviations

ICT: information and communication technology
TNBI: transgender, nonbinary, and intersex
