# Supplementary material for: Exploring Health Care Professionals’ Perspectives on Education, Awareness, and Preferences for Digital Educational Resources to Support Transgender, Nonbinary, and Intersex Care: Interview Study
Source: JMIR Med Educ. 2025 Mar 6;11:e67993. doi: 10.2196/67993 (PMC11926451; doi:10.2196/67993)
Supplement: Multimedia Appendix 1 [file mededu_v11i1e67993_app1.pdf]

Appendix A – Interview Guide

Age:
Geographic location:
Qualification/Education:
Position/role:
Years of experience in healthcare:
Experience in working with Transgenders and non-binary individuals and intersex:

Interview guide:

People

- **Question 1**: Can you describe your typical interactions with transgender and non-binary individuals in your healthcare practice?
- **Question 2**: Do you feel adequately trained in cultural competency regarding gender diversity and their specific needs?
- **Question 3**: What type of training or resources do you think would be most beneficial?

Workflow and Communication

- **Question 4**: What are the common challenges you encounter in establishing and maintaining connectivity with patients?
    - **Sub-question a**: Are there any specific communication barriers or misunderstandings that commonly arise?
    - **Sub-question b**: Can you share a specific situation where you felt additional support or resources would have improved your interactions?
- **Question 5**: What are your experiences with digital health platforms in fostering patient engagement and connectivity?
- **Question 6**: How do these platforms impact patient-provider communication and relationship-building?

Internal Organizational Policies, Procedures, and Culture

- **Question 7**: How do current internal policies and procedures support or hinder healthcare professionals in providing care for transgender, non-binary, and intersex individuals?
- **Question 8**: Are there any specific challenges or considerations that should be addressed to ensure seamless integration?

External Rules and Regulations, and Pressures

- **Question 9**: Are there any gaps in regulatory support that influence your approach to providing care to transgender, non-binary, and intersex individuals?

Clinical Content

- **Question 10**: What type of educational resources would assist you in better understanding the unique healthcare needs of transgender and non-binary individuals?
- **Question 11**: In what ways do you think such a tool could enhance the overall effectiveness of healthcare services for this population

Hardware and Software

- **Question 12**: Have you used any eHealth tools or resources aimed at assisting healthcare professionals in addressing challenges during interactions with transgender, non-binary, and intersex individuals?
- **Question 13**: What features or functionalities do you believe would be most beneficial in an educational digital tool designed for healthcare professionals working with transgender, non-binary, and intersex individuals? And why?
- **Question 14**: How do you envision integrating an eHealth tool into your current workflow when interacting with transgender and non-binary patients?

Human-Computer Interaction

- **Question 15**: How important is the ease of use and accessibility in an eHealth tool intended for healthcare professionals?
- **Question 16**: Do you believe additional training or support would be necessary for healthcare professionals to effectively utilize an eHealth tool for working with transgender, non-binary, and intersex patients?
- **Question 17**: What aspects of user experience do you consider crucial for effectively incorporating an eHealth tool into your practice?

System Measurement and Monitoring

- **Question 18**: In what ways do you think such a tool could enhance the overall effectiveness of healthcare services for this population?
